# Exploring the frontiers of research co-production: the Integrated Knowledge Translation Research Network concept papers

**DOI:** 10.1186/s12961-019-0501-7

**Published:** 2019-11-25

**Authors:** Ian D. Graham, Chris McCutcheon, Anita Kothari

**Affiliations:** 1 University of Ottawa, Faculty of Medicine, Ottawa Hospital Research Institute, Ottawa, Canada; 2 Ottawa Hospital Research Institute, Ottawa, Canada; 3 Western University, School of Health Studies, Ottawa, Canada

**Keywords:** Integrated knowledge translation, research co-production, engaged scholarship, participatory research, collaborative research

## Abstract

Research co-production is about doing research with those who use it. This approach to research has been receiving increasing attention from research funders, academic institutions, researchers and even the public as a means of optimising the relevance, usefulness, usability and use of research findings, which together, the argument goes, produces greater and more timely impact. The papers in this cross BMC journal collection raise issues about research co-production that, to date, have not been fully considered and suggest areas for future research for advancing the science and practice of research co-production. These papers address some gaps in the literature, make connections between subfields and provide varied perspectives from researchers and knowledge users.

Research co-production, sometimes referred to by such terms as participatory research, engaged scholarship, Mode 2 of knowledge production, collaborative research or integrated knowledge translation (IKT), is about conducting research with those who use it. Research co-production is a model of collaborative research, where researchers work in partnership with knowledge users (comprising patients and caregivers, clinicians, policy-makers, health system leaders and others) who identify a problem and have the authority or ability to implement the research recommendations [1]. As noted by Gagliardi et al. [2], IKT appears to increase researcher understanding of the research user context and needs, thereby enhancing the relevance of the generated research, and at the same time increase knowledge-user understanding of the research process, awareness of the research, and appreciation for how and when it can be applied.

Research co-production is promoted by funders and interested parties as a means of achieving research impact. The expectation is that the collaboration of researchers and knowledge users generates research that is particularly relevant, useful, useable and used. Research co-production is an appealing approach to addressing the ethical imperative of rapidly increasing the use of known effective healthcare innovations and decreasing over-use of ineffective ones. For others, it is about the democratisation of science and the right of citizens, who are taxed to pay for research, to participate in and influence the entire research process, not to just be considered for their role as passive research participants or subjects [3]. Other motivations are the desire to improve the quality of research which is believed to happen with inclusion of knowledge users by increasing researcher understanding of the issue, solutions and context, and partnering with knowledge users for political or strategic reasons [4].

Research co-production is not a new concept. It could be argued that participatory research, as espoused first by Kurt Lewin in the 1940s [5, 6] and then by Paulo Freire in the 1970s [7], was one of the first research traditions to focus on co-production. In Canada, while representing a very small proportion of national health research funding, the concept has been officially part of the health research ecosystem since the late 1990s, when funding programmes requiring inclusion of knowledge users as co-applicants were first launched [8]. Research co-production in health has been globally gaining interest. The funding of health research co-production is now taking place around the world. For example, in the United States, the Veteran Administration Quality Enhancement Research Initiative (QUERI) [9] and the National Institute on Disability, Independent Living, and Rehabilitation Research (NIDILRR) [10] encourage stakeholder engagement in research and the Patient-Centered Outcomes Research Institute [11] only funds research co-produced with patients and other stakeholders. The Australian Academic Health Centres [12–14], Dutch Academic Collaborative Centres [15], United Kingdom Academic Health Science Centres [16], United Kingdom Collaborations for Leadership in Applied Health Research and Care (CLAHRC), now known as Applied Health Centres [17, 18], all promote greater knowledge user participation in research and are premised on the theory that partnerships between universities/researchers and healthcare entities will increase the relevance and impact of health research. More evidence of the recognition accorded to research co-production is the emergence of what is being called ‘engagement science’, a field that investigates the methods for, and practice of, engagement, the development of evidence-based approaches or guiding frameworks for engagement, and the application of these resources to guide meaningful engagement of non-traditional stakeholders in research [19]. A recent series of papers on research co-production in the prestigious journal *Nature* further signals the growing attention this approach is receiving within the research community [20].

In 2015, the Canadian Institutes of Health Research approved funding for a 7-year foundation grant to contribute to building the science base for health research co-production or IKT, as it is referred to in Canada. This programme of research, known as the Integrated Knowledge Translation Research Network (IKTRN), comprises more than 30 knowledge-user experts (e.g. health research funders, health charities, regional health authorities and other organisations), over 40 IKT experts, a dozen knowledge translation/implementation science experts, and over 25 trainees from nearly 50 organisations in six countries (Canada, United States of America, England and Scotland, South Africa, Australia, Ireland) [21]. Kothari et al.’s definition of IKT (or research co-production) is, “*a model of collaborative research, where researchers work with knowledge users who identify a problem and have the authority to implement the research recommendations*” is the one adopted by the IKTRN [1]. The IKTRN also distinguishes between knowledge users (those who would make decisions or take actions based on study findings) and stakeholders (those with an interest in the research but who would not themselves directly act on the findings). While recognising that there are many research engagement frameworks that conceptualise a continuum of knowledge user engagement in research, typically ranging from more passive communication with knowledge users through to full partnership (researchers and knowledge users sharing power and decision-making), the IKTRN focuses on co-production in research collaborations where the researchers and the knowledge users aspire to regard themselves as equal partners. The goals, objectives and outputs of this research programme are described in the IKTRN’s research programme protocol, which is the first paper in this cross-journal collection [8].

Wanting to advance thinking and discussion on the science and practice of research co-production, in 2017, the IKTRN launched a call among Network members for critical concept papers that would begin defining areas of research co-production, advancing understanding of research co-production and focus for further research efforts, and provide an opportunity to generate discussion within the research community about research co-production. Some members of the network also offered empirical papers about research co-production they were working on. We believe the result to be a collection of innovative, thoughtful and timely papers about the theory, ethics, methods, evaluation and impact of research co-production as well as patient engagement and research co-production. This collection considers some of the key issues currently facing the science and practice of research partnerships, and collectively begins to identify elements of a research agenda for research co-production. For example, some papers consider how a research co-production approach relates to:

- Research methods (e.g. ethnography [22], community-based participatory research [23], evaluation of IKT [24])
- Indigenous health research [25]
- Global health governance [26]
- Patient engagement in research [27, 28]
- Creating impact [29]

Other papers include:

- A protocol for five scoping and systematic reviews on areas of research co-production [30]
- A review of what research funders around the world do to support knowledge translation and research co-production [31]
- A multiple case study of knowledge user participation in cancer health services research [32]

Many of these papers raise issues about research co-production that, to date, have not been fully considered and suggest areas for future research for advancing the science and practice of research co-production. These papers address some gaps in the literature, make connections between subfields, and provide varied perspectives from researchers and knowledge users.

In the Fall of 2018, the IKTRN brought together the authors of these papers to advance our thinking about these issues and to start charting what a research co-production research agenda might look like.

It is our hope that, collectively, these papers will inform, provoke thought and discussion, and generate interest in the concept and practice of research co-production.

## Data Availability

Not applicable.

## Abbreviations

IKT: integrated knowledge translation
IKTRN: Integrated Knowledge Translation Research Network
